# Dexamethasone inhibits the HSV-*tk*/ ganciclovir bystander effect in malignant glioma cells

**DOI:** 10.1186/1471-2407-5-32

**Published:** 2005-04-02

**Authors:** Pierre A Robe, Minh Nguyen-Khac, Olivier Jolois, Bernard Rogister, Marie-Paule Merville, Vincent Bours

**Affiliations:** 1 Department of Neurosurgery, University of Liège, Liège, Belgium; 2 Center for Biomedical Integrated Genoproteomics, University of Liège, Liège, Belgium; 3 Center for Molecular and Cellular Neuroscience, University of Liège, Liège, Belgium; 4 Department of Histology, University of Liège, Liège, Belgium

## Abstract

**Background:**

HSV-tk/ ganciclovir (GCV) gene therapy has been extensively studied in the setting of brain tumors and largely relies on the bystander effect. Large studies have however failed to demonstrate any significant benefit of this strategy in the treatment of human brain tumors. Since dexamethasone is a frequently used symptomatic treatment for malignant gliomas, its interaction with the bystander effect and the overall efficacy of HSV-TK gene therapy ought to be assessed.

**Methods:**

Stable clones of TK-expressing U87, C6 and LN18 cells were generated and their bystander effect on wild type cells was assessed. The effects of dexamethasone on cell proliferation and sensitivity to ganciclovir were assessed with a thymidine incorporation assay and a MTT test. Gap junction mediated intercellular communication was assessed with microinjections and FACS analysis of calcein transfer. The effect of dexamethasone treatment on the sensitivity of TK-expressing to FAS-dependent apoptosis in the presence or absence of ganciclovir was assessed with an MTT test. Western blot was used to evidence the effect of dexamethasone on the expression of Cx43, CD95, CIAP2 and Bcl_XL_.

**Results:**

Dexamethasone significantly reduced the bystander effect in TK-expressing C6, LN18 and U87 cells. This inhibition results from a reduction of the gap junction mediated intercellular communication of these cells (GJIC), from an inhibition of their growth and thymidine incorporation and from a modulation of the apoptotic cascade.

**Conclusion:**

The overall efficacy of HSV-TK gene therapy is adversely affected by dexamethasone co-treatment *in vitro*. Future HSV-tk/ GCV gene therapy clinical protocols for gliomas should address this interference of corticosteroid treatment.

## Background

Herpes Simplex virus thymidine kinase gene (HSV-*tk*) suicide gene therapy has lately been used in a variety of cancer models to sensitize replicating cells to the antiviral drug ganciclovir (GCV) [1-3]. In this paradigm, tumor cells transfected with the HSV-*tk *gene become able to mono-phosphorylate ganciclovir, a nucleotide analog. Ganciclovir monophosphate is subsequently bis and tri-phosphorylated by cellular kinases and is then incorporated in the DNA of replicating cells, blocking the cell cycle and inducing apoptosis [1,4,5]. Cells that are transfected and exposed to ganciclovir can also kill adjacent, untransfected cells by the so-called bystander effect. In most tumor types, the bystander effect relies on the transfer of phosphorylated ganciclovir molecules between cells via their gap junctions [6,7], although other mechanisms have been described in some models [8-11].

Given their resistance to conventional treatments and their confinement to the brain, malignant gliomas have undergone a variety of human trials of HSV-*tk *gene therapy. The initial enthusiasm has however declined as a large, multicenter phase 3 clinical study failed to demonstrate any survival benefit for patients treated with HSV-TK gene therapy [12]. Efforts are now directed at improving the protocols, especially by modifying the vectors of gene distribution in tumor cells [13], the antiviral drugs [14] and the bystander effect [15-17]. A few papers have also pointed out that some concomitant treatments reduce the benefit of HSV-*tk*/ ganciclovir gene therapy [18]. In this view, and given the widespread use of corticosteroids in the symptomatic treatment of malignant gliomas patients, we have assessed the effect of dexamethasone on the bystander effect in this type of cancer.

## Methods

### Cells and culture conditions

Rat C6 malignant glioma cells (ATCC # CCL-107) were grown in Dulbecco's modification of Eagle's medium supplemented with 3% fetal bovine serum and penicillin (DEM-3%FBS). C6-TK cells were obtained as described previously [16] and maintained in DEM-3% FBS supplemented with 500 μM geneticin. Human U87 (ATCC # HTB-14) and LN18 cells (a gift from Prof. N de Tribolet) were grown in RPMI 1640 medium supplemented with 10% fetal bovine serum and penicillin (RPMI-10%FBS). U87-TK and LN18-TK cells were obtained as for C6-TK cells [17] and kept in RPMI-10% FBS supplemented with 500 μM geneticin.

### Gap junction intercellular communication

Cells grown for 48 hours in the presence or absence of dexamethasone were loaded either with CMTMR, a gap-junction impermeant dye or with calcein, which diffuses freely through gap junctions. Cells were then mixed for 4 hours, and the percentage of CMTMR-tagged cells having incorporated calcein after this period – a direct correlate of GJIC- was determined by FACS analysis of 10,000 cells [17]. For microinjection experiments, cells were grown on polyornithine-coated glass coverslips to confluence and then treated or not with dexamethasone (10^-6 ^M) for 24 hours. The coverslips were then placed in the culture chamber of a Zeiss^® ^fluorescence microscope perfusion chamber of a dedicated Zeiss fluorescence microscope, bathed with EA01 buffer (NaCl 137 mM, KCl 5.7 mM, CaCl2 1.8 mM, D-Glucose 22.2 mM, Hepes 10 mM), and injected with a solution of Lucifer Yellow for 30 seconds as previously described [16]. The number of dye-colored cells was then counted one minute after the end of the injection.

### Western blots

Western blots were performed as previously described [17]. Rabbit polyclonal antibodies to connexin 43 (Zymed, San Francisco, CA), CIAP-2, BCL_XL _and FAS/CD95 (Santa Cruz Biotechnologies, Heidelberg, Germany) were employed for antigen detection while monoclonal antibodies to tubulin (Santa Cruz) or actin (Roche, Mannheim, Germany) were used to assess loading homogeneity. Immunodetection was carried with HRP-coupled secondary antibodies to mouse (Sigma-Aldrich, Bornem, Belgium) or rabbit (Amersham, Uppsala, Sweden) antibodies and a chemoluminescent peroxidase substrate (Pierce, Rockford, IL).

### Assessment of the bystander effect

Given proportions of TK^- ^and TK^+ ^cells were seeded in 96-wells plates at a final density of 10,000 cells/well and grown for five days in the presence or absence of ganciclovir (2 – 10 μM) and/or dexamethasone (1 μM). Media were replaced every 48 hours. Cell survival was then assessed with a 3(4, 5-dimethylthiazol-2-yl)-2, 5-diphenyltetrazolium (MTT) test as described previously [16]. With this test, the cellular viability in each well is directly correlated to its optical density measured at a wavelength of 650 nm (OD_650_). Results are expressed as the ratio of the OD_650 _of ganciclovir-treated cells to that of untreated control wells (set as 100 %). Experiments were run 3 times in triplicate in the presence or the absence of dexamethasone, and results are given as mean ± SD for each condition.

### Sensitivity of TK^+ ^cells to GCV

C6-TK5 cells were seeded in duplicate in 24-wells plates at a density of 100,000/well and grown for 5 days in the presence of increasing concentrations of ganciclovir. They were then harvested and counted on a hemocytometer using the trypan blue exclusion test. Experiments were run three times. Results are shown as the percentage (± SD) of live cells with respect to live cells in control, untreated wells. For U87-TK and LN18-TK cells, the sensitivity of cells to GCV (10 μM) after 5 days in culture was assessed in the presence or absence of dexamethasone (1 μM) with a MTT test (see previous paragraph for details).

### Cell growth and survival

C6, LN18 and U87 cells were grown for 5 days in the presence or absence of dexamethasone and cell viability was assessed with a MTT test. For thymidine incorporation experiments, C6, LN18 and U87 cells were grown in triplicate in 24-wells plates in culture medium supplemented ^3^H-thymidine (4 μCi/ml, Pharmacia-Amersham, Rosendaal, The Nederlands) for 48 hours. The incorporation was stopped after several PBS washes by digestion of the cells in 1 ml of 0.1 N NaOH, and ^3^H-thymidine activity was recorded with a Wallac^® ^1400 scintillation counter. Results were normalized with respect to the protein content of each culture well as assessed with the method of Bradford [19]. Results are shown as the mean of 3 independent experiments ± SD.

### CD95-induced cell death

100,000 cells were seeded in triplicate in 96-wells culture plates and cultured for 24 hours in the presence of soluble FAS-Ligand (sFAS-L, 2 ng/ml, Alexis, Lausen, Switzerland), dexamethasone (1 μM), ganciclovir (10 μM) and of a combination of these drugs. Cell viability was then assessed with an MTT test. Experiments were run three times in triplicate. Results are expressed as the ratio of cell viability in treated wells to that in untreated wells, ± SD.

### Statistical analysis

Statistical analyses were performed as stated in the 'Results' section with the Statview software version 5.0 (SAS Institute, Cary, NC).

## Results

### Dexamethasone decreases the bystander effect in C6, LN18 and U87 cells

**Figure 1:**
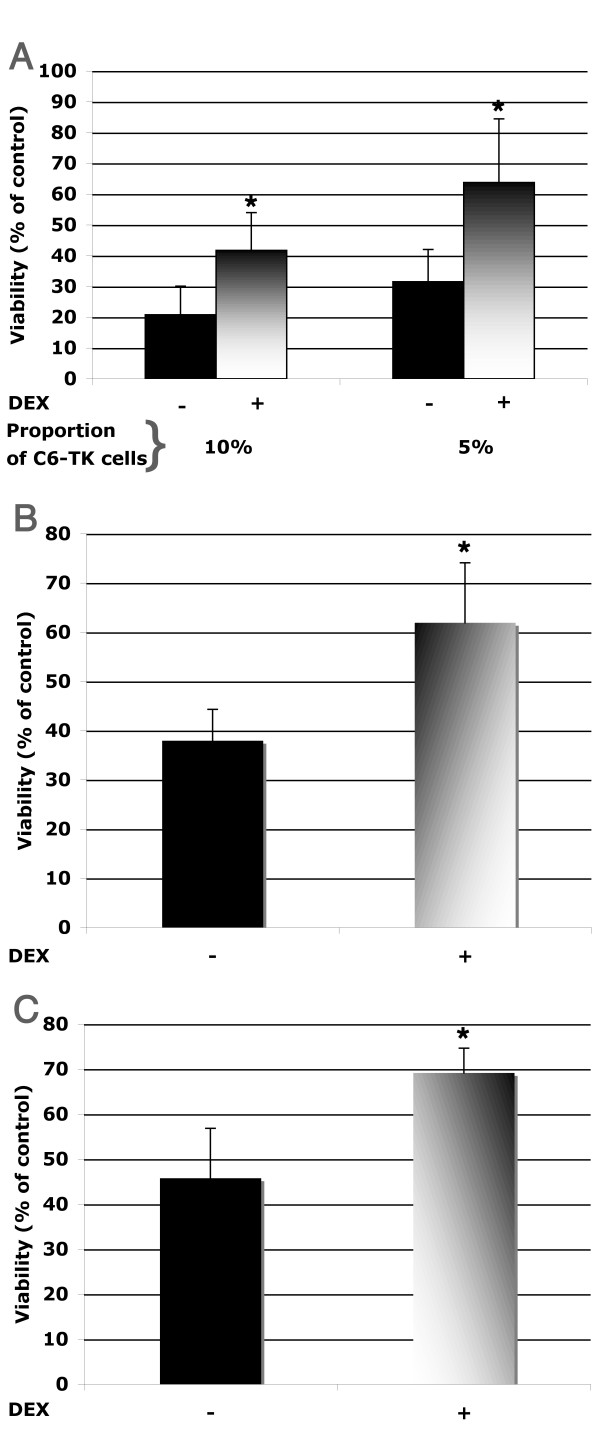
**Effect of dexamethasone on the bystander effect**. **(A) **The viability of C6 cells mixed with 10 % (left) or 5 % (right) of C6-TK cells and treated with ganciclovir (2 μM) for 5 days is shown in the presence (+) or absence (-) of dexamethasone (1 μM, DEX). **(B) **Viability of LN18 cells mixed with 20 % LN18-TK cells and grown for 5 days with ganciclovir (10 μM) in the presence (+) or absence (-) of DEX. **(C) **Viability of U87 cells mixed with 10 % U87-TK cells and grown for 5 days with ganciclovir (10 μM) in the presence (+) or absence (-) of DEX. For each condition, the viability in the absence of ganciclovir was set as 100 percents. (*: *P *< 0.05, Student's t-test)

C6 cells were mixed with 5 or 10 % of C6-TK cells, and treated with GCV (2 μM). In the absence of dexamethasone (DEX^-^), the overall cell viability after 5 days in culture was respectively 20.7 ± 9.3 % and 31.5 ± 10.4 % of that of controls. It increased to 41.7 ± 12.3 % and 63.8 ± 20.6 % respectively in the presence of 1 μM dexamethasone (DEX^+^, p < 0.05, Student's t-test, Figure 1A). For LN18 cells grown in the presence of 20% LN18-TK cells and treated with 10 μM GCV, the overall survival of DEX^- ^cells was 37.94 ± 6.37 % of that of controls but increased to 61.93 ± 12.19 % in DEX^+ ^wells (p < 0.05, Student's t-test, Figure 1B). Similarly, the overall viability of a U87 cells mixed with 10% of U87-TK cells and exposed to 10 μM GCV increased from 45.76 ± 11.1% (DEX^-^) to 69.16 ± 5.51 % in DEX^+ ^conditions (p < 0.05, Student's t-test, Figure 1C).

### Dexamethasone reduces the gap junction intercellular communication (GJIC) of glioma cells

**Figure 2:**
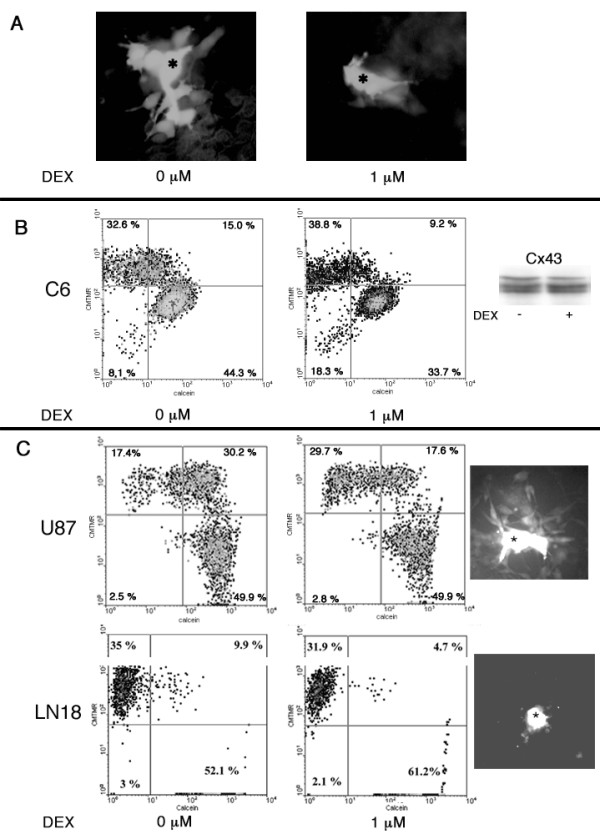
**Effect of dexamethasone on gap junction intercellular communication**. **(A) **Appearance of confluent cultures of C6 cells after iontophoresis of Lucifer Yellow in one single cell (*) in the absence (left) or presence (right) of dexamethasone (DEX). Photomicrographs are representative of the typical appearance of such injected cells. **(B) **FACS analysis of calcein dye transfer between rat C6 cells in the presence (right) or absence (left) of DEX. The GJIC is directly correlated with the percentage of CMTMR^+^/Calcein^+ ^cells (upper right quadrants, see text for details). This experiment is representative of 3 independent experiments each performed on 10,000 cells. A Western blot of Cx43 in C6 cells is shown (left: control conditions, right, DEX treatment. See text for details) **(C) **FACS analysis of calcein dye transfer between human U87 (above) and LN18 (below) cells in the presence (right) or absence (left) of DEX. The rightmost pictures illustrate the basal GJIC of both cell types in confluent cultures after iontophoresis of Lucifer Yellow in a single cell (*, control conditions).

In iontophoresis experiments, Lucifer Yellow dye diffused to 10.3 ± 10.6 C6 cells (n = 33) per injected cell in control conditions, and to 7.5 ± 6.5 cells (n = 13) in the presence of 10^-6^M of dexamethasone (NS tendency, Student's t-test, mean ± SD, Figure 2A). In order to assess the GJIC in much larger cell populations, we analyzed the diffusion of calcein dye in C6, LN18 and U87 cells by flow cytometry (FACS). This technique reproducibly demonstrated a reduction of GJIC following treatment with dexamethasone (1 μM). For example, when CMTMR^+ ^C6 cells were mixed with calcein-loaded, CMTMR^- ^C6 cells, the percentage of CMTMR^+ ^cells that incorporated calcein after 4 hours in culture decreased from 15.25 ± 0.911 % in control conditions (DEX^-^) to 10.7 ± 1.875 % in the presence of 1 μM dexamethasone (n = 3, p = 0.0179, Student's t-test). Using a polyclonal antibody against rat connexin 43, we did not observe any modulation of the expression and phosphorylation of Cx43 in C6 cells treated with dexamethasone for 24 hours (Figure 2B). A similar inhibition of GJIC occurred in LN18 and U87 cells treated with 1 μM dexamethasone for 24 hours (Figure 2C). LN18 and U87 cells respectively exhibited a very low and high level of GJIC in control conditions (Figure 2C).

### Dexamethasone reduces the sensitivity of TK^+ ^cells to ganciclovir

**Figure 3:**
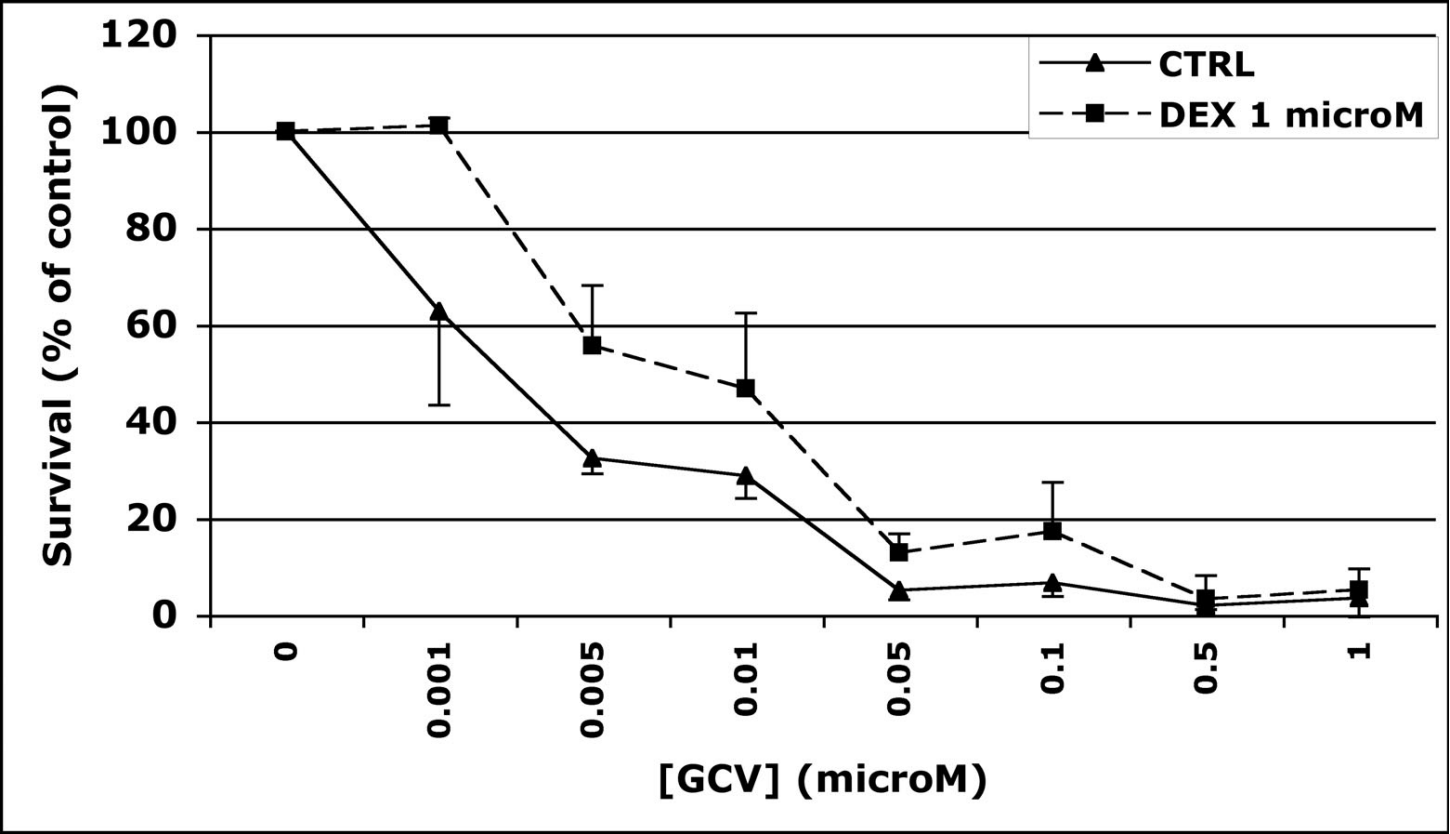
**Sensitivity of C6-TK cells to ganciclovir**. The sensitivity of C6-TK cells is shown in control conditions (▲) and in response to treatment with 1 μM dexamethasone (■). Results are expressed as the percentage of living cells in treated conditions at various concentrations of ganciclovir with respect to ganciclovir-free cultures, and are shown as the mean of 3 independent experiments.

The curve that represents the survival of C6-TK cells exposed to increasing concentrations of ganciclovir for 5 days was significantly shifted to the right in the presence of 1 μM dexamethasone (p < 0.05, multiple ANOVA, Figure 3). Similarly, the viability of LN-TK and U87-TK cells treated with 10 μM GCV for 5 days increased 2.35-fold and 1.47-fold respectively in DEX^+ ^conditions as compared with DEX^- ^conditions (n = 3, p < 0.05, Student t-test). The viability of wild-type C6, U87 and LN18 cells remained unaffected by ganciclovir (data not shown).

### Dexamethasone alters glioma cells proliferation

**Figure 4:**
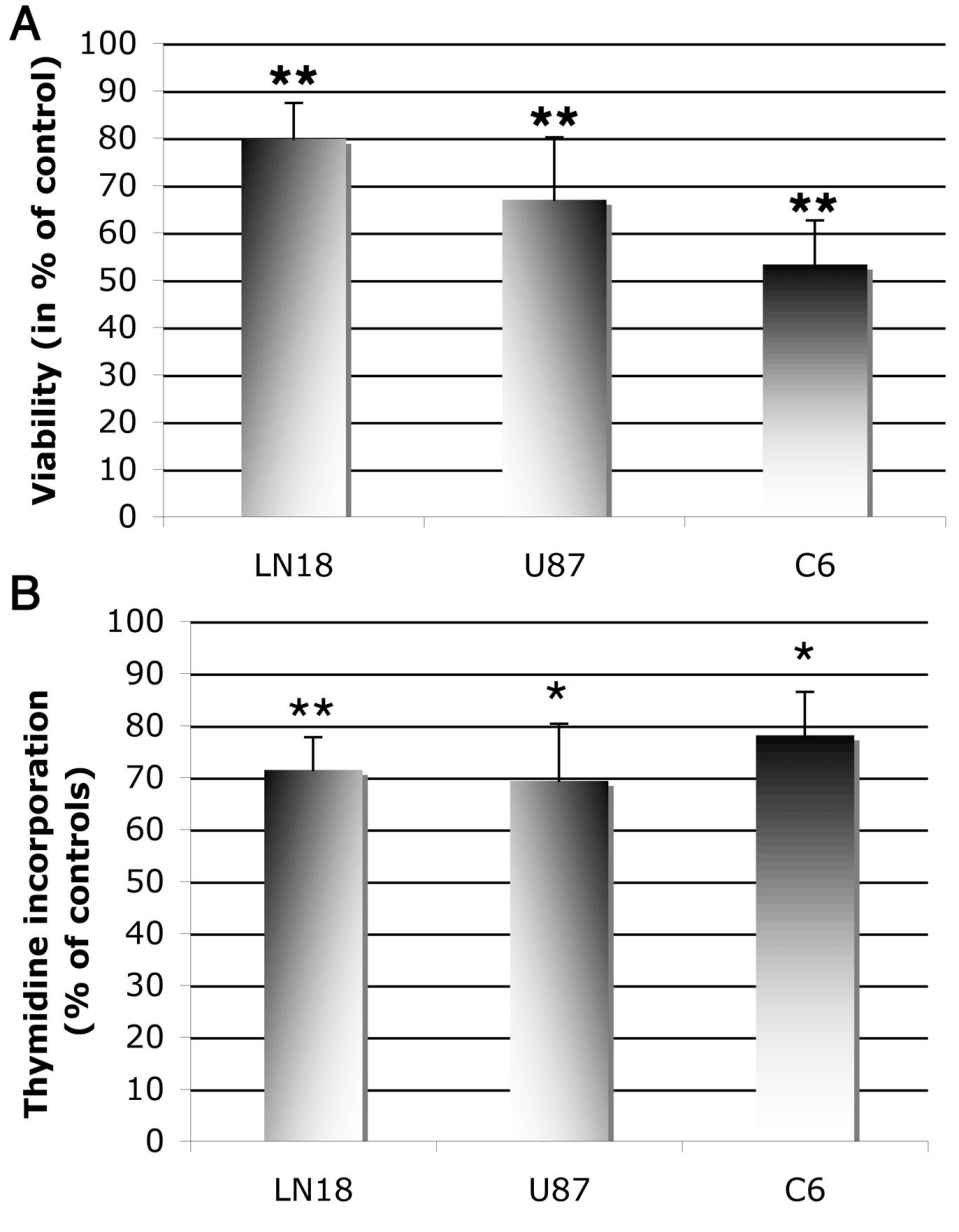
**Cell growth and thymidine incorporation**. **(A) **The total viability of LN18, U87 and C6 cells grown for 5 days with 1 μM dexamethasone (DEX) was determined by way of an MTT test. For each condition, the viability in the absence of DEX was set as 100 percents (n = 3, mean ± SD). **(B) **The incorporation of ^3^H-thymidine in LN18, U87 and C6 cells grown for 48 hours in the presence of DEX is shown (n = 3, mean ± SD). For each condition, the incorporation in the absence of DEX was set as 100 percents. (*: *P *< 0.05; ** *P *< 0.005, Student's t-test).

The viability of wild-type C6, U87 and LN18 cells after 5 days in culture (logarithmic growth phase) was reduced by respectively 46.75 ± 9.3 %, 33.1 ± 13.25 % and 20.6 ± 7.6 % in the presence of DEX (n = 3 for each condition, p < 0.005 each, One sample t-test, Figure 4A). DEX also decreased the incorporation of ^3^H-thymidine in C6, U87 and LN18 cells by 21.9 ± 8.4 %, 30.7 ± 11 % and 28.5 ± 6.3% respectively (n= 3 for each condition, p < 0.05 each, Student's t-test, Figure 4B).

### Dexamethasone alters CD95-triggered apoptosis in glioma cells

**Figure 5:**
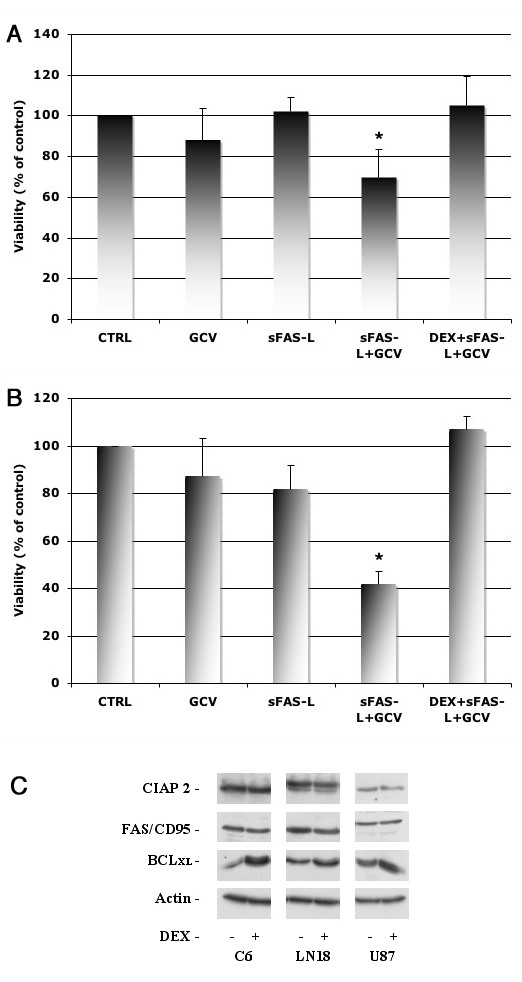
**Effect of dexamethasone on FAS/CD95-triggered apoptosis**. **(A) **Viability of C6-TK cells after 24 hours of growth in the presence of GCV, sFAS-L, sFAS-L + GCV and DEX + sFAS-L + GCV, assessed with a MTT test. This viability was significantly altered in cells treated with sFAS-L and GCV and this sensitization was abolished by DEX. **(B) **Viability of LN18-TK cells after 24 hours of growth in the presence of GCV, sFAS-L, sFAS-L + GCV and DEX + sFAS-L + GCV. This viability was significantly altered in cells treated with sFAS-L and GCV and this effect was abolished by DEX. For both cell types, the viability in the absence of any drug was set as 100 percents. (*: *P *< 0.05, ANOVA). **(C) **Western blot analysis of the expression of CIAP-2, FAS/CD95 and BCL_XL _in C6, LN18 and U87 cells following treatment with DEX (1 μM).

GCV (10 μM) significantly sensitized LN-TK and C6-TK cells but not U87-TK cells to the toxic action of soluble FAS-L (sFAS-L, 2 ng/ml). This effect was abolished by DEX (Figure 5A and 5B, and data not shown). Dexamethasone (1 μM × 24 hours) did not alter the expression of FAS/CD95 and of the apoptosis inhibitor CIAP-2 in LN18, C6 and U87 cells with these cells, as evidenced by Western blot. The expression of the anti-apoptotic protein BCL_XL _however slightly but reproducibly increased in C6 and LN18 cells (Figure 5C).

## Discussion

In this study, we assessed the influence of dexamethasone on the bystander effect of HSV-TK/ ganciclovir gene therapy in gliomas. Dexamethasone is indeed commonly used for the symptomatic treatment of brain tumor patients, and the bystander effect is considered a major contributor to the efficacy of type of suicide gene therapy [20,21].

We found that a concentration of 1 μM, i.e. within its therapeutic range [22], dexamethasone significantly inhibited the bystander effect on C6, LN18 and U87 glioma cells co-cultured with small proportions (5–20 %) of C6-TK, LN18-TK and U87-TK cells. This finding is important, since 10 % of TK-expressing cells represent the threshold at which C6 tumors can be cured by HSV-TK suicide gene therapy [2] and since usually less than 5 % of cells are transfected *in vivo *with currently available vectors [12,13].

The *in vitro *bystander effect in glioma cells depends on the transfer of phosphorylated ganciclovir molecules trough gap junctions [23,7]. Dexamethasone at 10^-6 ^M moderately but significantly reduced the GJIC of C6, U87 and LN18 cells, which is a know mechanism through which drugs decrease the bystander effect [16]. This moderate reduction of GJIC by DEX in cells that exhibit a low (LN18), medium (C6) and high (U87) level of GJIC [17] and originate from humans and a rodent, is consistent with previously published results [24]. This modulation of GJIC in C6 cells did not appear to result from a reduced expression or a differential phosphorylation of connexin 43, the major connexin protein of malignant astroglial cell [25,26]. We cannot exclude that some Cx43 phosphorylation changes were not evidenced with the antibody we used. It is also possible that the effect of DEX results from a modification of cell adhesion molecules or other connexin-interacting proteins as has been suggested for other effectors [27,28].

It also inhibited by 23–53 % the log-phase growth of all three types of glioma cells after 5 days in culture, as well as their incorporation of ^3^H-thymidine. Of note, dexamethasone has also been shown to decrease glioma size in humans *in vivo*, an effect that is well-known to clinicians but is usually transient and lasts for a few weeks only [29]. Since the incorporation of the nucleotide analogue triphosphoganciclovir in replicating DNA strands is required for its toxicity both in TK-expressing and bystander cells, this anti-proliferative effect of dexamethasone may contribute to its inhibition of the bystander effect. Such a mechanism has been described for another inhibitor of DNA replication, Ara-C, that reduces the bystander effect without altering the GJIC in C6 cells [16]. It is somewhat surprising that dexamethasone decreased both cell proliferation and GJIC in our experiments. A decrease of connexin expression and GJIC is indeed usually associated with an enhanced proliferation of tumor cells [30,18]. We however did not observe any dexamethasone-induced alteration of Cx43 expression on our Western blots, and the enhancement of GJIC by itself is less likely to control the growth of tumor cells than is the expression of connexin proteins [31,25]. Finally, glucocorticoids are also known to alter cell proliferation via GJIC-independent mechanisms such as cell cycle blockade [32] and alter the PI3K-Akt pathway [33].

GCV treatment has been shown to induce apotosis in TK-expressing and bystander cells, an effect that notably implies both ligand-dependent and independent CD95 receptor activation [34-36]. We found that ganciclovir indeed sensitized C6-TK and LN18-TK cells to the lethal action of soluble sFAS-L, and this effect was abolished by dexamethasone. This drug did neither reduce the expression of FAS/CD95 protein expression nor induce that of apoptosis inhibitor CIAP-2 in our hands. This latter result contrasts with the findings of Webster *et al*. who observed an induction of CIAP-2 by dexamethasone [37]. We however observed an enhanced expression of BCL_XL_, a known modulator of FAS-dependent apoptosis [38] by dexamethasone in our three glioma cell lines, as also described by Gorman *et al*. [39].

The reasons why ganciclovir failed to enhance the toxicity of sFAS-L on U87-TK cells remain unknown and are beyond the scope of the present work, as is the thorough assessment of the effects of dexamethasone on HSV-TK/GCV-induced apoptosis. Our results nevertheless demonstrate that dexamethasone reduces the bystander effect of HSV-TK/ ganciclovir gene therapy via at least three different mechanisms in rodent and human gliomas in vitro, i.e. a modulation of GJIC, thymidine incorporation and apoptotic pathways.

The *in vivo *significance of our results must also still be validated. Indeed, although the effects of dexamethasone on HSV-*tk *gene therapy have partially been studied *in vivo *on the 9L animal model of glioma [40,41], none of these studies specifically addressed the bystander effect. In one of these studies however [40], dexamethasone tended to reduce the survival of animals inoculated with TK-expressing 9L cells and treated with ganciclovir. Despite a small number of animals in the GCV (n = 9) and in the GCV + dexamethasone (n = 10) treatment arms, and although most of the observations in the control group were censored, this difference almost reached significance (cited *P *= 0.057). In this study, only 10 % of the animals treated with dexamethasone + GCV remained alive 60 days after tumor implantation (termination of the observation period) versus 55 % of the animals treated with GCV alone, a result that is consistent with our *in vitro *data. Given the fundamental role of the bystander effect in clinical situations [12], and since the ultimate evaluation of the effects of dexamethasone on the bystander effect will eventually depend on human clinical trials, the design of upcoming protocols should carefully address this potential drug interaction.

## Conclusion

The overall efficacy of HSV-TK gene therapy is adversely affected by dexamethasone co-treatment *in vitro *through a modulation of apoptosis and gap junction intercellular communication. Although additional *in vivo *research is necesseray to confirm this finding, future HSV-tk/ GCV gene therapy clinical protocols for gliomas should address this interference of corticosteroid treatment.

## Competing interests

The author(s) declare that they have no competing interests.

## Authors' contributions

PR is responsible for the generation of TK-expressing cells, microinjection experiments and apoptosis experiments and contributed to the Western blots, cell proliferation assays and evaluation of the bystander effect. He is also responsible with VB for the initiation of the project. MTN contributed to the Western blots, cell proliferation assays and evaluation of the bystander effect. OJ is responsible for the FACS experiments. BR contributed to the Western blots, cell proliferation assays and evaluation of the bystander effect and the design of the experiments. MPM contributed to the design of the experiments. All authors read and approved the final manuscript.

## Pre-publication history

The pre-publication history for this paper can be accessed here:
